# Enhanced homologous recombination by the modulation of targeting vector ends

**DOI:** 10.1038/s41598-020-58893-9

**Published:** 2020-02-13

**Authors:** Shinji Hirotsune, Hiroshi Kiyonari, Mingyue Jin, Kanako Kumamoto, Kayo Yoshida, Miki Shinohara, Hitomi Watanabe, Anthony Wynshaw-Boris, Fumio Matsuzaki

**Affiliations:** 1 Department of Genetic Disease Research, Osaka City University, Graduate School of Medicine, Asahi-machi 1-4-3, Abeno, Osaka, 545-8585 Japan; 2 Animal Resource Development Unit, Genetic Engineering Team, Division of Bio-Function Dynamics Imaging, RIKEN Center for Life Science Technologies, 2-2-3 Minatojima-Minamimachi, Chuou-ku, Kobe, 650-0047 Japan; 3 Laboratory Animal Science, Osaka City University, Graduate School of Medicine, Asahi-machi 1-4-3, Abeno, Osaka, 545-8585 Japan; 4 Institute for Protein Research, Osaka University 3-2 Yamadaoka, Suita, Osaka, 565-0871 Japan; 5 Present Address: Faculty of Agriculture, Department of Advanced Bioscience, Kindai University, 3327-204 Nakamachi, Nara-city, Nara 631-8505 Japan; 6 Laboratory of Integrative Biological Science, Institute for Frontier Life and Medical Sciences, Kyoto University, Kyoto, 606-8507 Japan; 7 Department of Genetics and Genome Sciences, Case Western Reserve University, School of Medicine, University Hospitals Case Medical Center 10900 Euclid Avenue, BRB731, Cleveland, Ohio 44106-4955 USA; 8 RIKEN Center for Developmental Biology, 2-2-3 Minatojima-Minamimachi, Chuou-ku, Kobe, 650-0047 Japan

**Keywords:** Genetic engineering, Genetic engineering, Genetic engineering, Genetic engineering

## Abstract

The field of genome editing was founded on the establishment of methods, such as the clustered regularly interspaced short palindromic repeat (CRISPR) and CRISPR-associated protein (CRISPR/Cas) system, used to target DNA double-strand breaks (DSBs). However, the efficiency of genome editing also largely depends on the endogenous cellular repair machinery. Here, we report that the specific modulation of targeting vectors to provide 3′ overhangs at both ends increased the efficiency of homology-directed repair (HDR) in embryonic stem cells. We applied the modulated targeting vectors to produce homologous recombinant mice directly by pronuclear injection, but the frequency of HDR was low. Furthermore, we combined our method with the CRISPR/Cas9 system, resulting in a significant increase in HDR frequency. Thus, our HDR-based method, enhanced homologous recombination for genome targeting (eHOT), is a new and powerful method for genome engineering.

## Introduction

The generation of targeted genome-modified animals provides an important approach for investigating gene functions in pathogenesis and gene therapy in humans. With conventional gene-editing methods, mutations are introduced through homology-directed repair (HDR) in embryonic stem (ES) cells. Usually, chimeric animals are generated by the injection of gene-targeted ES cells into wild-type (WT) blastocysts. Subsequent mating of chimeric animals produces mice carrying the targeted gene modification^1^. Alternative methods exist in which DNA or mRNA that encode site-specific zinc finger nucleases (ZNFs) or transcription activator-like effector nucleases (TALENs) are directly injected into a fertilized egg to introduce DNA double-strand breaks (DSBs) at a specified locus, and these methods have been developed in a variety of species to target specific genomic sites^2–6^. Recently, type II bacterial clustered regularly interspaced short palindromic repeat (CRISPR) and CRISPR-associated protein (CRISPR/Cas) system-based gene-targeting tools have been applied for multiplex genome editing^7–9^. Indeed, the CRISPR/Cas system has been demonstrated to drive both nonhomologous end joining (NHEJ)-based gene disruption and HDR-based precise gene editing^10,11^. Furthermore, the CRISPR/Cas system is currently being applied to inducible multiplex gene regulation, genome-wide screens and cell fate engineering^12^. Although the CRISPR/Cas system is an excellent method for genome modification, there are still limits to its application, including off-target effects and low gene replacement efficiency.

HDR and classical nonhomologous end joining (C-NHEJ) are central cellular pathways involved in the repair of DSBs. In addition to these central pathways, other error-prone repair systems, known as alternative nonhomologous end joining (A-NHEJ) and microhomology-mediated end joining (MMEJ), are genetically independent from C-NHEJ. In C-NHEJ, the Ku heterodimer binds to DSB ends to protect DNA from extensive resection and unwinding. In MMEJ, DSB ends are resected, and the exposed DNA fragments that exhibit microhomology anneal. The repair process is followed by overhang removal, gap filling DNA repair and ligation. These systems often generate small sequence changes near the DSB site and promote random integration during targeted mutation processes^13,14^. In contrast, HDR promotes genetic changes by using an uninjured homologous region as a template to restore correct information that was lost during a DSB. This allows HDR to be mostly error-free^15^. HDR is composed of three pathways: presynapsis, synapsis, and postsynapsis^15^. First, in presynapsis, a recombination-proficient DNA fragment carrying resected DNA ends is generated either by endonucleolytic incision or genotoxic stress caused by replication problems or the surrounding environment. Second, in synapses, a physical connection (also called a displacement-loop) is generated by the invasion of recombinogenic strands into an intact homologous DNA template, which constitutes new hybrid DNA strands. Third, in postsynapsis, HDR is completed by ligation to form duplex DNAs after endonucleolytic cleavage of either crossed strands or noncrossed strands^15^. In postsynapsis, although the three subpathways of HDR are distinct, they share the same initial steps, in which a 3′ overhanging tail is generated by the resection of DSB ends. The generation of a 3′ overhanging tail is an essential step for assembling the Rad51 filament, which is followed by a search for homology and an invasion by the recombinogenic DNA strand^15^. Thus, we hypothesized that the DNA ends of a targeting vector would likely affect the efficiency of HDR-based gene targeting. To test this hypothesis, we investigated whether exonuclease treatment would improve HDR-based gene targeting. We discovered that treatment with T7 exonuclease, which catalyzes the removal of 5′ mononucleotides from duplex DNA in the 5′ to 3′ direction, provides a 3′ overhanging tail on the targeting vector that increases the efficiency of HDR.

## Results

### Targeting frequency is governed by the shape of the vector ends

**Figure 1:**
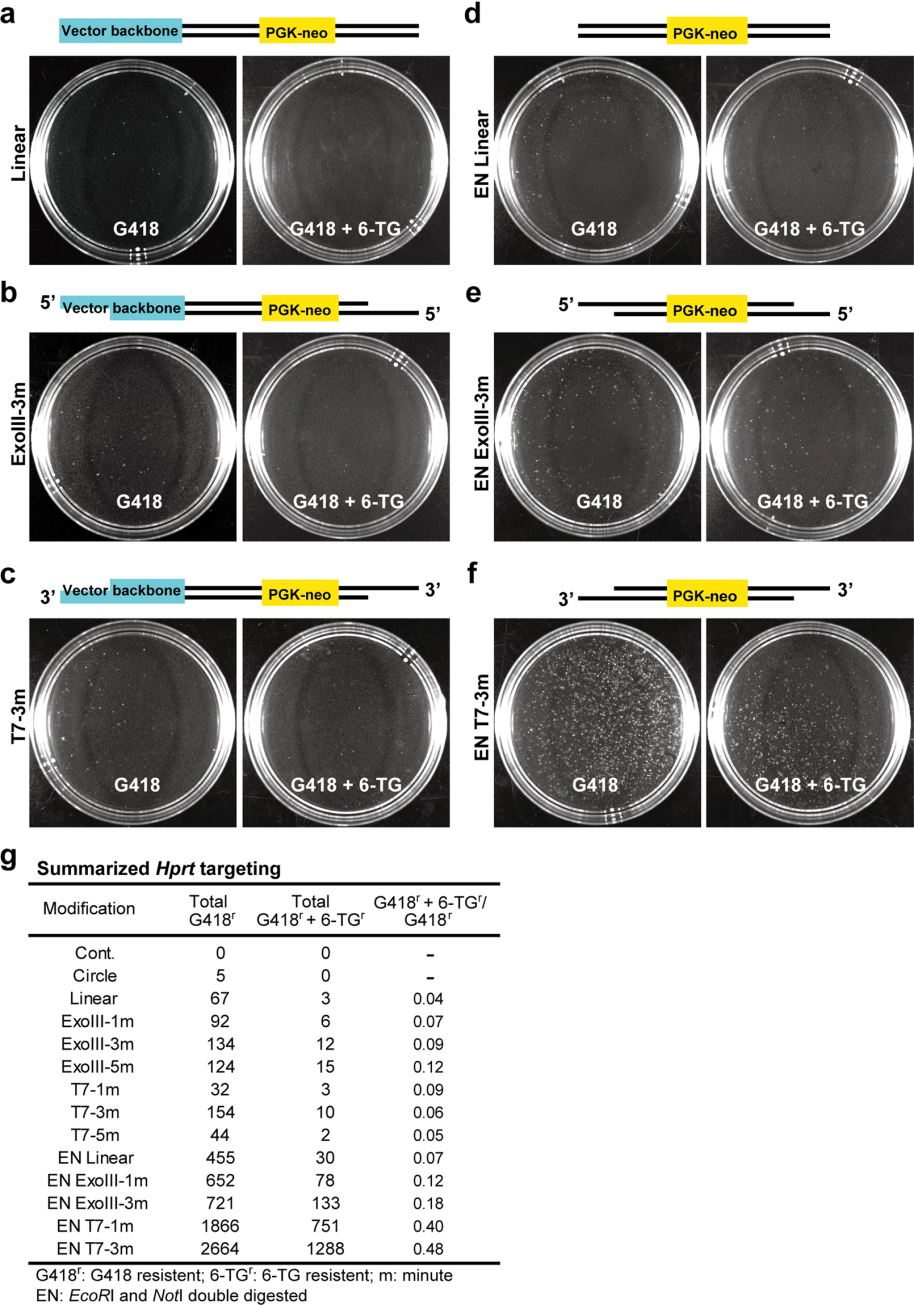
*Hprt* gene targeting. (**a–c**) Targeting vectors were linearized by *Not*I digestion only (**a**), *Not*I digestion followed by ExoIII treatment to provide a unilateral 5′ overhangs of the *Hprt* gene (**b**), or T7 treatment to provide a unilateral 3′ overhangs of the *Hprt* gene (**c**). (**d–f**) *Not*I and *Eco*RI double digestion was performed to remove the vector backbone (**d**) or was followed by ExoIII treatment (**e**) or T7 treatment (**f**). The ES cell colonies in (**a–f**) were subjected to selection by G418 only for 7 days (left side) or to further G418 + 6-TG selection (right side) for the next 7 days, as indicated. The schematic illustrations in the upper side of each image set represent the linearized targeting vectors carrying blunt ends (**a,d**), 5′ overhangs (**b,e**), or 3′ overhangs (**c,f**) in each experiment. (**g**) Summary of G418 only or additional G418 + 6-TG selection for 7 days is shown. The *Hprt* targeting vector carrying bilateral 3′ overhangs increased the number of G418- and 6-TG-resistant clones, suggesting that bilateral 3′ overhangs greatly facilitate HR frequency.

To examine the effect of the vector ends on HDR efficiency, we performed analyses in mouse ES cells by specifically assessing the gene targeting frequency and integration patterns of the vectors; we did this by using a series of replacement vectors designed to target the hypoxanthine phosphoribosyltransferase gene, *Hprt*^16,17^. In XY ES cells, *Hprt* is hemizygous, so *Hprt*-targeted clones can be identified by 6-thioguanine (6-TG) selection for loss of *Hprt* function^16,18^. Exonuclease enzymes cleave the nucleotides at the ends of DNA molecules. Exonuclease III (ExoIII) acts in the 3′ to 5′ direction, catalyzing the removal of 3′ mononucleotides from duplex DNA, whereas T7 acts on the opposite strand in the 5′ to 3′ direction. We first determined the enzyme activities using SYBR Green I. After the digestion of an *eGFP* empty vector by various restriction enzymes, SYBR Green I was intercalated into the *eGFP* empty vector, and then the vector was treated with ExoIII or T7. The reduction in fluorescence intensity was correlated with the length of either the 5′ or 3′ overhangs and thus allowed us to estimate the lengths of the overhanging vector ends. Based on our results, we estimated that ExoIII and T7 removed, on average, 100 nucleotides/min and 150 nucleotides/min, respectively (Supplementary Fig. 1a,b). We further confirmed the lengths of the overhangs that were used in this study by the same method, which revealed that the enzyme activity was consistent regardless of which vector was used (Supplementary Fig. 1c–h). We constructed a vector containing a *neo* cassette flanked by sequences homologous to the *Hprt* gene (Supplementary Fig. 2a), and then we used ExoIII or T7 to introduce a variety of modifications to the vector ends (Fig. 1a–f). We first examined the frequency of HDR-mediated gene targeting to the *Hprt* gene and other error-prone repair systems, including C-NHEJ and MMEJ, by selection with G418 for one week; then, double selection was performed using G418 and 6-TG (HDR-mediated gene targeting of the *Hprt* gene) for an additional week. Linearization of the targeting vector at one end seemed to promote efficient integration (Fig. 1a,g), as previously reported^19,20^. We then transfected cells with the ExoIII-treated targeting vector, which provided a 5′ overhanging tail or the T7-treated vector, which provided a 3′ overhanging tail. These modifications moderately enhanced integration frequency, suggesting that vector end overhangs are critical factors that govern HDR and other pathways by facilitating the DNA damage response (DDR) (Fig. 1b,c,g and Supplementary Fig. 2b). Next, we applied double digestion with restriction enzymes to expose both of the homologous regions that were flanked by the neo cassette. Exposure of both vector ends clearly increased the integration frequency (Fig. 1d,g). We further applied ExoIII or T7 treatment to these homologous regions. To our surprise, these treatments increased the frequency of integration (Fig. 1e–g). The bilateral 3′ overhangs obtained with the T7 treatment increased the frequency of integration (G418-resistant clones), especially in *Hprt* targeting, where the frequency was 3-fold higher than it was following the ExoIII treatment (Fig. 1f,g). In particular, T7 treatment in homologous regions increased the number of G418-resistant clones by 39-fold (67 colonies to 2664 colonies; Fig. 1g) and increased the number of G418 and 6-TG double-resistant clones 429-fold (3 colonies to 1288 colonies; Fig. 1g). Therefore, the proportion of G418 and 6-TG double-resistant clones in the G418 resistant clones increased 12-fold (4% to 48%; Fig. 1g). Our results appeared to augment the total activity of the DSB repair machinery, leading to an increase in random integration, but HDR was even more clearly enhanced. The G418 and 6-TG double-resistant clones may have contained background mutations, such as spontaneous mutations in the *Hprt* gene or the accidental loss of the X chromosome. Previous reports have demonstrated that *Hprt* mutations can mainly be attributed to replacement by a targeting vector^21^; thus, we believed that the increase in G418 and 6-TG double-resistant clones could be attributed to the facilitation by HDR. We named this method “enhanced HDR for genome targeting” (eHOT). HDR and non-HDR systems, including C-NHEJ and MMEJ, compete to repair DSBs, but the balance between them differs widely depending on DNA damage pattern and cell cycle stage^22,23^. Our data showed that the presence of an optimal 3′ overhanging tail may promote HDR-mediated DNA repair.

**Figure 2:**
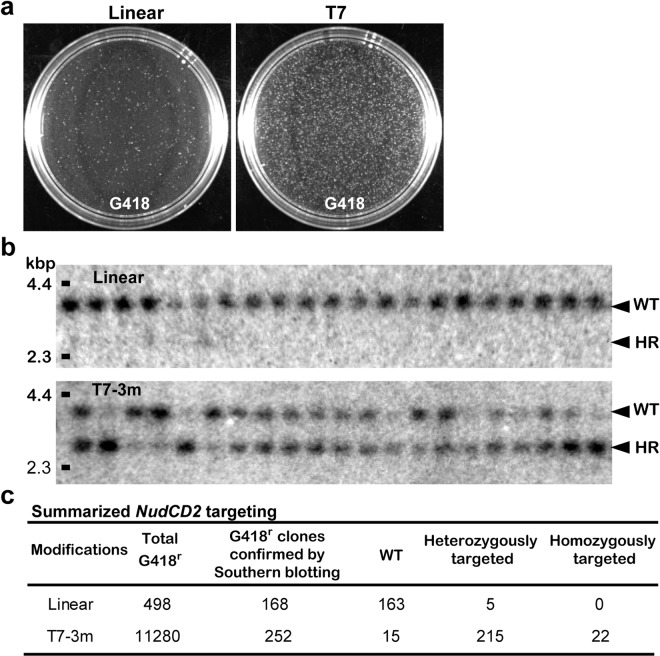
*NudCD2* gene targeting. (**a**) ES cell colonies after 7 days of selection with G418 are shown. Treatment with T7 increased the number of G418-resistant colonies. (**b**) Southern blotting analyses of G418-resistant clones after *Kpn*I digestion using a flanking probe (Supplementary Fig. 2c) are shown. A *Hin*dIII digest of lambda DNA was used as a size marker (left side). The targeted allele is shorter than the WT allele due to the introduction of a novel *Kpn*I site. (**c**) Summary of genotype analysis of G418-resistant clones. Treatment with T7 increased the number of homologous recombinant clones.

To determine whether this method was applicable to other loci, we targeted the NudC domain-containing protein 2 gene (*NudCD2)*. This *nudC* gene, a member of the nud gene family, regulates nuclear migration in *Aspergillus nidulans*^24^. NudF is also a member of this family, whose mammalian orthologue *Lis1* encodes a protein essential for neuronal migration^25,26^. In the cell cycle, NUDCD2 localized first at the centrosomes and then at the spindle poles and kinetochores during interphase and mitosis, respectively. This localization pattern is similar to LIS1 and cytoplasmic dynein, and depletion of NUDCD2 significantly affects LIS1 and dynein function. We performed gene targeting of *NudCD2* (Supplementary Fig. 2c) in ES cells using the newly established eHOT method. The presence of an optimal 3′ overhanging tail in the targeting vector for *NudCD2* increased the number of G418-resistant colonies 23-fold (498 colonies to 11280 colonies) compared to that of the same vector that was only linearized (Fig. 2a,c). We next examined homologous recombinants by Southern blotting using an external probe. Surprisingly, 237 clones of the 252 G418-resistant colonies were homologous recombinants (Fig. 2b,c; and Supplementary Fig. 2d–h), and the proportion of homologous recombinant clones out of the G418-resistant clones was increased 31-fold (3% to 94%). Furthermore, 22 of those 237 homologous recombinant clones were homozygous for the target mutation (Fig. 2b,c; and Supplementary Fig. 2f–h). Curiously, HDR in the *NudCD2* locus was more efficient than it was at the *Hprt* locus. The degree of HDR efficiency appears to be dependent on the intrinsic properties of each locus. These results from *Hprt* and *NudCD2* indicate that targeting vectors carrying 3′ overhanging ends enhanced integration into the genome and the frequency of HDR. Thus, eHOT technology is applicable to other loci in ES cells.

### Examination of the DSB response in fertilized mouse eggs

These data prompted us to address whether eHOT could be used to directly modify the genomes of fertilized eggs via pronuclear injection. To apply eHOT to fertilized mouse eggs, it was essential to determine whether the HDR machinery was active. We first examined the DSB response in fertilized mouse eggs using the radiomimetic drug neocarzinostatin (NCS), which induces DSBs^27^. H2AX is phosphorylated on the 139th serine residue at the loci of DNA damage^28^. Termed γ-H2AX^29^, this type of damage is the first step in recruiting and localizing DNA repair proteins for both NHEJ and HDR. One of the key proteins that accumulates at the damaged site is 53BP1^30,31^, which is a key factor in NHEJ. When the resection of damaged DNA ends commences, NHEJ is halted, and the proteins involved in HDR are recruited. These proteins include Rad51, which builds helical nucleoprotein filament structures and facilitates annealing of the complementary DNA strands^32,33^. Therefore, 53BP1 and Rad51 signals can be used to indicate potential NHEJ-mediated repair and HDR-mediated repair sites, respectively, at the site of DNA damage, which is marked by γ-H2AX positive foci. Therefore, we examined the colocalization of 53BP1/γ-H2AX and Rad51/γ-H2AX in mouse embryonic fibroblast (MEF) cells and in ES cells after treatment with NCS. NCS treatment significantly increased the frequency of 53BP1/γ-H2AX- and Rad51/γ-H2AX-positive foci in both MEF cells and ES cells (Supplementary Fig. 3a–f). Curiously, ES cells appeared to display a relatively high frequency of 53BP1/γ-H2AX- and Rad51/γ-H2AX-positive foci under untreated conditions (Supplementary Fig. 3d–f), which may have been due to the rapid progression of the cell cycle in ES cells.

**Figure 3:**
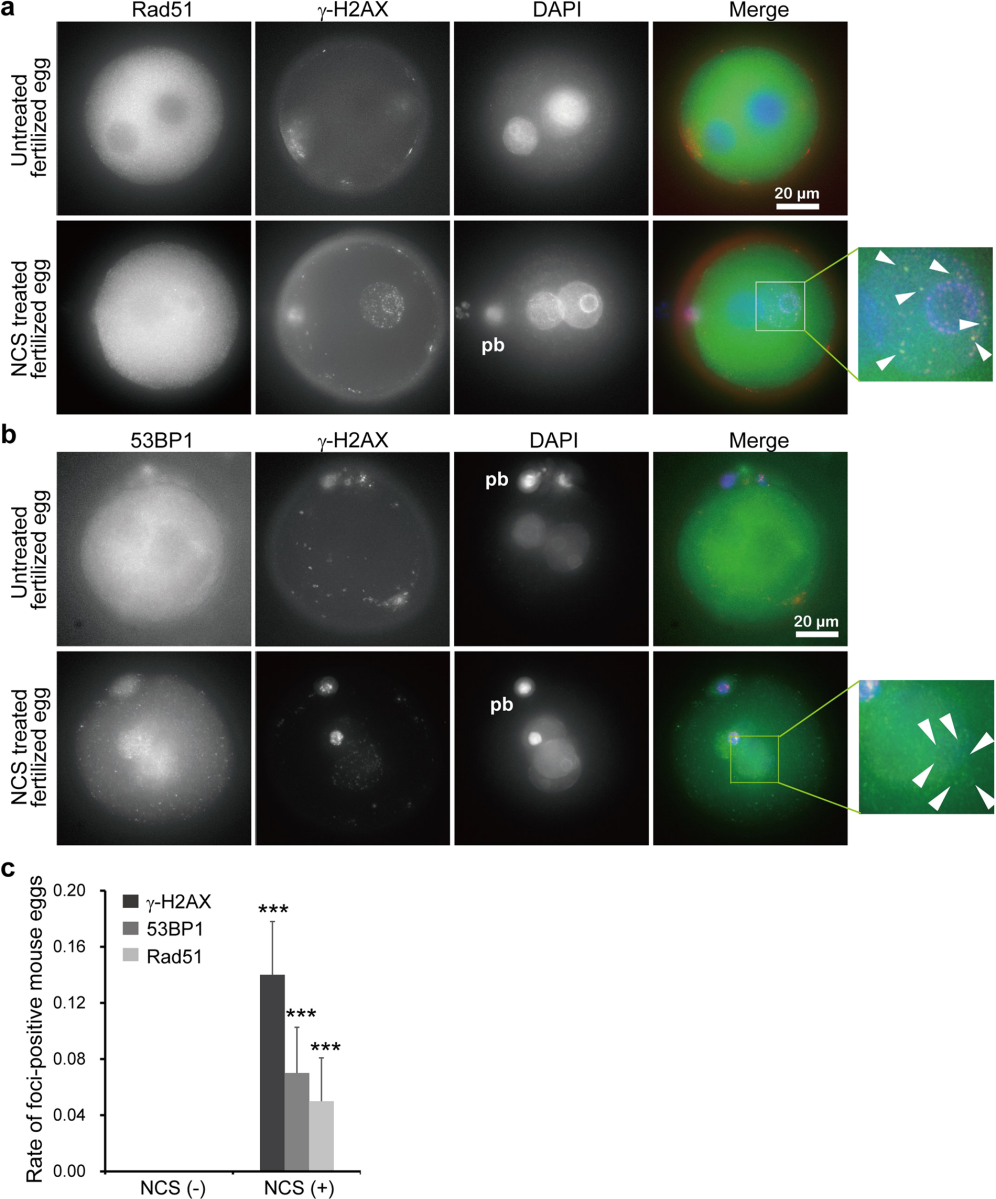
Examination of DNA repair after DSB introduction in fertilized mouse eggs. DNA repair was probed by analyzing the increases in γ-H2AX, Rad51 and 53BP1 expression after NCS treatment. (**a**,**b**) Colocalization of γ-H2AX with Rad51 (**a**) or with 53BP1 (**b**) was investigated after NCS treatment. Areas surrounded by a rectangle in (**a,b**) are shown enlarged at right. White arrowheads indicate colocalization of γ-H2AX with Rad51 or with 53BP1. (**c**) Statistical analysis was performed for colocalization of γ-H2AX with Rad51 or with 53BP1 (*n* = 42 for nontreated control zygotes, *n* = 116 for NCS-treated fertilized eggs). The background activity levels of γ-H2AX, Rad51 and 53BP1 in the zygotes were very low. In contrast, γ-H2AX, Rad51 and 53BP1 were highly active in the polar bodies without NCS treatment. *P*-values were calculated using Student’s *t*-test, mean ± s.e., ***P < 0.001. Scale bar: 20 μm. pb: polar body.

As demonstrated by γ-H2AX foci formation after exogenous DSB induction, mouse oocytes from preovulatory follicles contain DSBs^34,35^. Although a relatively small amount of ataxia telangiectasia mutated (ATM) is expressed in full-grown oocytes compared to that of growing oocytes or blastocysts, H2AX was phosphorylated in these oocytes despite the limitation of ATM activation^34^. Indeed, DSBs induced by the bleomycin family of drugs delay the progression of meiosis and activation of the anaphase promoting complex (APC/C)^34,35^. Foreign DNA can be introduced into the mouse genome by direct microinjection of DNA into the pronuclei of fertilized single-cell mouse embryos^36^; this process is mainly mediated by NHEJ, indicating that the DSB repair machinery is functional in fertilized mouse eggs. After 1 h of treatment with NCS, we found that 53BP1/γ-H2AX and Rad51/γ-H2AX were clearly colocalized in fertilized mouse eggs. Although the level of γ-H2AX in fertilized eggs was relatively lower than it was in MEF cells or ES cells, the frequency of Rad51/γ-H2AX foci (Fig. 3a,c and Supplementary Fig. 3g) was similar to that of 53BP1/γ-H2AX foci (Fig. 3b,c and Supplementary Fig. 3h), indicating that HDR and NHEJ are active in fertilized mouse eggs. Curiously, without NCS treatment, the frequency of Rad51/γ-H2AX and 53BP1/γ-H2AX foci was extremely low in fertilized mouse eggs (Fig. 3).

### Direct gene targeting in fertilized mouse eggs by pronuclear injection

**Figure 4:**
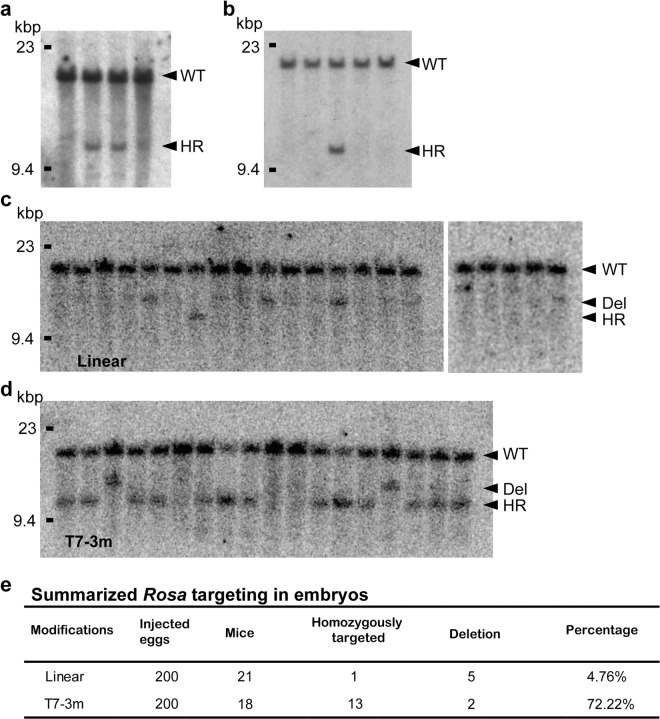
Gene targeting of *Rosa26* and *NudCD2* by pronuclear injection. (**a**) Southern blotting analysis of pups born after targeting the *Rosa26* locus by pronuclear injection. Selected candidates and controls were analyzed. Polymorphisms were detected after *Eco*RI digestion with a flanking probe (Supplementary Fig. 4a). The intensity of the targeted allele was weaker than that of the WT allele, suggesting that these pups were genetic mosaics. (**b**) Southern blotting analysis of pups born after targeting the *NudCD2* locus by pronuclear injection. One selected candidate and control were analyzed. Polymorphisms were detected after *Apa*I digestion with a flanking probe (Supplementary Fig. 4e). *Hind*III digest of lambda DNA was used as a size marker (left side). (**c**) Southern blotting analysis of embryos after targeting the *Rosa26* locus by pronuclear injection of T7 untreated *Rosa26* vector with CRISPR/Cas9-mediated DSB. Polymorphisms were detected after *Eco*RI digestion with a flanking probe (Supplementary Fig. 4a). (**d**) T7treated *Rosa26* vector was used. Arrows indicate WT, simple deletion by NHEJ, and HDR. (**e**) Summary of Southern blotting analysis of embryos after targeting the *Rosa26* locus by pronuclear injection with CRISPR/Cas9-mediated DSB.

The presence of HDR in fertilized mouse eggs prompted us to try direct gene targeting of the *Rosa26* locus by pronuclear injection of targeting vectors carrying 3′ overhangs. *Rosa26* is a locus used for constitutive, ubiquitous gene expression in mice^37^. We generated a targeting vector for *Rosa26* (Supplementary Fig. 4a), removed the vector backbone, treated the vector with T7, and then injected this treated targeting vector into *C57BL/6* fertilized eggs. We first injected the unmodified vector into 504 eggs and obtained 117 pups. We next injected the modified vector into another set of 459 eggs and obtained 110 pups. We performed genotyping analysis by Southern blotting using a flanking probe (Supplementary Fig. 4a) and found two homologous recombinants following injection with the modified vector (Fig. 4a and Supplementary Fig. 4c,d). In contrast, we found no homologous recombinants when the unmodified vector was used (Supplementary Fig. 4b,d). The intensity of the Southern blot derived from the targeted allele was lower than that of the WT allele (Fig. 4a), suggesting that the *Rosa26*-targeted mice produced by microinjection of plasmids into pronuclei were genetic mosaics. This result implies that in these lines, integration of the injected DNA occurred after the first round of DNA replication in fertilized eggs. To address whether eHOT is able to modify another locus, we generated a targeting construct against *NudCD2* (Supplementary Fig. 4e) and treated fertilized eggs in a similar fashion. Targeting vectors for *NudCD2* carrying 3′ overhangs were injected into *FVB* fertilized eggs. We first injected the unmodified vector into 360 eggs in total and obtained 97 pups. We next injected the modified vector into another 213 eggs and obtained 41 pups. We performed genotyping analysis by Southern blotting using a flanking probe (Supplementary Fig. 4e) and found one homologous recombinant in the modified vector group (Fig. 4b and Supplementary Fig. 4g,h). In contrast, we found no homologous recombinants when the unmodified vector was used (Supplementary Fig. 4f,h). The intensities of the Southern blot derived from the targeted allele were similar to those of the WT allele, suggesting that HDR-mediated integration may have occurred during the first round of cell division. Although the frequencies of these homologous recombinants were not statistically significant, these results demonstrated that HDR-based genome modification could be achieved using targeting vectors with 3′ overhangs.

### Combining eHOT with CRISPR/Cas9

The CRISPR/Cas9 system is a valuable tool for genome engineering through the introduction of site-specific genomic DSBs. CRISPR/Cas9 has begun to be combined with HDR to introduce controlled genetic modifications, such as codon replacement or insertion of reporter genes by HDR, with exogenous targeting vectors serving as repair templates. However, HDR efficiency is limited by the presence of a competing repair pathway that is far more efficient, NHEJ. To enhance the frequency of HDR, global inhibition of key NHEJ factors has been the most widely studied strategy^38–40^. Although these approaches are effective in increasing the frequency of HDR, further improvements are required to achieve more practical HDR-based genome engineering. Thus, we applied eHOT to HDR after the introduction of DSBs by the CRISPR/Cas9 system. We introduced two separate DSBs into the *Rosa26* locus by the injection of crRNAs with the donor template. Southern blotting analysis demonstrated that the targeting vectors for *Rosa26* carrying 3′ overhangs significantly increased the frequency of HDR (13/18) compared to that of the control template (1/21) (Fig. 4c–e). We further confirmed the presence of a specific modification by PCR wherein we used primers that detected the *GFP* sequence (Supplementary Fig. 4i). In contrast, there was no significant difference in NHEJ-dependent deletion (5/21 and 2/18). Thus, we concluded that modifying vectors to carry 3′ overhangs is a valuable method for generating HDR recombinants in fertilized mouse eggs.

Despite its enhancement through the use of CRISPR/Cas9-mediated targeting or other modified nucleases, HDR-based knock-in is still relatively inefficient^41^. Intensive efforts have been made to improve the efficiency of HDR-based knock-in by the following methods: suppressing the NHEJ pathway using small chemical compounds^38,39,42^, enhancing HDR via synchronizing cell cycles to the G2/M phase^43^, and increasing RAD51 expression by treatment with valproic acid^44^. Our method facilitated HDR by optimizing vector ends. In the first step of HDR, the 5′-ends of DSBs were resected to create 3′ overhangs. The overhangs served as both an ideal substrate for the assembly of proteins required for DNA strand invasion and a primer for new DNA synthesis. Preparation of targeting vectors carrying 3′ overhanging ends contributed to this critical step, thereby facilitating HDR. Furthermore, the introduction of DSBs to the targeting locus by the CRISPR/Cas9 system also elicits DSB repair machinery. Presumably, the combination of the introduction of DSBs in the genome and the optimization of vector ends towards HDR will achieve synergistic effects that enhance the knock-in process.

## Discussion

In this paper, we demonstrated that the use of targeting constructs carrying long 3′ overhangs facilitates the frequency of HDR, suggesting that the creation of 3′ single-stranded DNA (3′ ssDNA) overhangs is a rate-limiting step in HDR. Indeed, we succeeded in gene targeting by pronuclear injection of constructs with 3′ overhangs into fertilized eggs.

The hallmark of HDR is error-free recovery of genetic information using a homologous region as a template, which may have been lost during DSBs or in subsequent DNA processing. HDR begins with the resection of DSB ends from 5′ to 3′ to produce 3′ ssDNA. The overhanging strand invades into a homologous template strand to create a D-loop, in which the 3′ end of the broken chromosome pairs with an intact DNA side through binding of homologous regions. Invasion of these 3′ ssDNA overhangs into a homologous region is followed by new DNA synthesis from the invading end using the intact DNA as a template, which can then be repaired by several HDR-mediated pathways, such as double-strand break repair (DSBR) and synthesis-dependent strand annealing (SDSA). There is a series of sequential reactions, and the generation of 3′ ssDNA by resection from the 5′ to 3′ direction of the broken ends serves as the rate-limiting step; therefore, the generation of 3′ overhangs with appropriate lengths enhances HDR frequency.

Intriguingly, our results showed that the generation of 5′ overhangs also enhanced HDR frequency, though less efficiently than the generation of 3′ overhangs. This finding suggests that 5′ overhangs facilitate HDR through other mechanisms. In DSBR-mediated HDR, after strand invasion and synthesis of new DNA, the other end of a DSB can be captured to generate an intermediate with two Holliday junctions (HJs), which are crucial intermediates in HDR and are essential for the creation of crossover outcomes. In a previous report, it was shown that strand crossing mediated by eukaryotic recombinases also proceeds from the 3′ to 5′ direction, corresponding to the single-stranded substrate DNA^45^. The opposite polarity of recombinases also makes this mechanism suitable for the repair of DSBs. The increase in HDR frequency by 5′ overhangs may be explained by this alternative HDR mechanism. It is unclear why 3′ ssDNA overhangs more efficiently enhance HDR. One possibility is that single-stranded 3′ overhangs are common precursors for DSBR and SDSA. Another possibility is that 3′ ssDNA overhang-dependent HDR is somehow dominant over 5′ ssDNA overhang-dependent HDR.

The current view is that the cell cycle is a key regulator that directs repair towards either the HDR or NHEJ pathways^46^. Therefore, the status of the cell cycle is crucial for HDR-mediated genome modification. In a mouse fertilized egg, the first cell cycle is relatively long, and it reaches the two-cell stage 1.5 days postcoitum (dpc). The following four cell cycles average approximately 12 h each, resulting in 32-cell early blastocysts at 3.5 dpc^47^. Cell cycles from the initial blastocyst to the late blastocyst of the implantation stage (~120 cells) last approximately 24 h on average^48^. Here, we demonstrated the presence of Rad51 foci in fertilized eggs, which suggests the possibility of HDR-based genome modification. Indeed, we succeeded in HDR-based gene targeting by pronuclear injection, but the efficiency was not high. Therefore, further improvements in the transfection stage, to the transfection methods and to the DNA modifications (such as cytosine modifications) are essential for facilitating increased HDR frequency.

Technologies for genome editing and manipulation have been greatly advanced by the recent application of various strategies for targeted DSBs, including the CRISPR/Cas9 system. However, gene targeting requires a repair step after introducing DSBs, and the repair depends on either HDR or NHEJ. Not much attention has been paid to improving the efficiency of this step. In this study, we focused on this aspect of the gene-targeting strategy, and our technology, eHOT, achieved a major improvement in HDR efficiency. Although HDR is diverse among different organisms and cell types, the core steps of HDR are highly conserved across all organisms. Thus, our eHOT method will be applicable to a wide range of organisms. Furthermore, we demonstrated that this method can be used in combination with the CRISPR/Cas9 method to improve the efficiency of gene targeting. To enhance HDR, key factors involved in NHEJ, such as DNA-PKcs, Ku70, and DNA ligase IV, have been inhibited by widely used methods, such as shRNA-treatment, proteolytic degradation, or treatment with small molecules^38–40^. In HDR, the 5′-end of a DSB is resected to create ssDNA carrying a 3′ overhang. This overhang serves as both a substrate for the assembly of proteins involved in strand invasion and a primer for new DNA synthesis in HDR. Thus, our method facilitates HDR by preparing vector ends that are suitable for the HDR pathway. eHOT will be applicable for technically difficult challenges in the future, such as making humanized animals for studying important regions of the genome and extending the genomic applications of CRISPR/Cas9 engineering.

## Methods

The methods were carried out in accordance with the relevant guidelines. All experimental protocols were approved by the Gene Modification Experiments Safety Committee Modification Experiments Safety Committee Gene Modification Experiments Safety Committee of Osaka City University (authorization number OCU-142601) and Singapore (IRB-10051). All mouse experiments were performed with the approval of the Animal Care and Ethics Committee of Osaka City University (authorization number OCU-08033).

All animal experiments carried out in this study were also approved by the Animal Care Committee of the Institute for Frontier Life and Medical Sciences, Kyoto University, and conformed to institutional guidelines for the study of vertebrates.

### Targeting constructs

The targeting vectors used in this study contained the neomycin resistance cassette *pPNTNeo*^49^ inserted into exon 3 of *Hprt*. We modified the original *pPNTNeo* vector by introducing *Asc*I sites and *Sfi*I sites. In this modified *pPNTNeo* vector, the entire insert was demarcated by *Asc*I and *Sfi*I sites at its borders such that the entire insert could be cut out by digestion with either *Asc*I or *Sfi*I. The targeting vectors prepared from isogenic DNA were constructed from a genomic sequence of *Hprt* isolated from *129/Sv* mouse genomic DNA by PCR amplification. Schematics of the targeting vectors are shown in Supplementary Fig. 2a. Exon 3 and the neighboring introns of *Hprt* were replaced with the neomycin cassette. The targeting vector carried 3.4 kbp homologous regions of the *Hprt* gene on both sides. The *Not*I site of the *pPNTNeo* vector was used for simple linearization of the vector backbone carrying the *Hprt* targeting construct. This *Not*I-digested *Hprt* targeting vector was also used for further ExoIII treatment. For treatment with T7, *Not*I-digested constructs were treated with Klenow fragment to generate blunt ends prior to T7 treatment. The *Asc*I *or Sfi*I sites of the modified *pPNTNeo* vector were used to cut out the entire insert, and the vector was then treated with ExoIII or T7, respectively.

For gene targeting of *NudCD2* in ES cells, we used our modified *pPNTNeo* vector. The neomycin cassette *pPNTNeo* was inserted into the beginning of exon 1 of *NudCD2*, and the third *loxP* sequence was inserted into intron 2 (Supplementary Fig. 2c). *Not*I digestion was applied for simple linearization of the *NudCD2* targeting construct. To generate 3′ overhangs on both ends of the homologous region, the *NudCD2* targeting construct was digested with *Sfi*I, and T7 treatment was subsequently performed. For all targeting constructs, we applied phenol-chloroform extraction followed by ethanol precipitation to remove all enzymes used before transfection into ES cells.

### Transfection and cell culture

We initially examined the sensitivity of TC1 ES cells to selection media. The cells were sensitive to G418 (250 μg/ml, Roche Diagnostics, Basel, Switzerland) and 6-thioguanine (6-TG: 1 μg/ml, Sigma-Aldrich), and we found no obvious colonies after seven days of selection (Supplementary Fig. 2b). Each *Hprt* vector (25 μg) was linearized with *Not*I and transfected into TC1 ES cells as described previously^17^. The DNA concentrations of other modified vectors were adjusted to the *Not*I linearized targeting vector such that the number of DNA molecules per cell was the same for each electroporation. Just prior to electroporation, the ES cells were trypsinized, washed once with serum-free Dulbecco’s modified Eagle medium (DMEM), and resuspended in electroporation buffer (Opti-MEM, Thermo Fisher Scientific, MA, USA) at a concentration of 10^7^ cells per ml. Each targeting vector solution was added to 1 ml of cell suspension, mixed, and electroporated using a Gene Pulser II (600 V/cm, 25 μF: Bio-Rad, CA, USA). Following electroporation, cells were diluted with DMEM plus serum and plated onto mitomycin-treated Neo-resistant MEF feeder cells at a concentration of 10^6^ ES cells per 100-mm-diameter petri dish. Cells were fed fresh medium every other day. After 24 h of incubation in nonselective medium, the medium was supplemented with G418 (250 μg/ml). Cells were maintained under G418 selection for 7 days, followed by an additional 7 days of selection in G418 (250 μg/ml) plus 6-TG (1 μg/ml). At the end of this period, the number of surviving colonies was determined. For *NudCD2* targeting, 25 μg of each *NudCD2* vector was linearized with *Not*I and transfected into TC1 as described previously^17^. The T7-treated *NudCD2* targeting constructs were also transfected into TC1, and the DNA concentrations were adjusted such that the number of DNA molecules per cell during electroporation was the same as that of the *Not*I linearized targeting vector. Following electroporation, cells were plated on mitomycin-treated Neo-resistant MEF feeder cells at a concentration of 10^6^ ES cells per 100-mm-diameter petri dish. After 24 h of incubation with nonselective medium, G418 selection was performed. We also prepared plates in which the ES cells were further diluted (10^5^ ES cells per 100-mm-diameter petri dish and 10^4^ ES cells per 100-mm-diameter petri dish) for picking colonies. HDR clones were identified by Southern blotting analysis using a restriction polymorphism provided by a *Kpn*I site that was newly introduced into the targeted allele (Supplementary Fig. 2c: WT allele 3.1 kbp, targeted allele 2.5 kbp).

### Estimation of ExoIII and T7 activity

To examine the enzyme activity of ExoIII or T7, we utilized real-time quantitative PCR using a fluorescent reporter, SYBR Green I (Thermo Fisher Scientific, MA, USA). SYBR Green I has been used in a variety of quantitative dsDNA detection and analysis techniques. Briefly, the *eGFP* empty vector (Clontech) was linearized with *Eco*RI (leaving a 5′ protruding end), *Sma*I (leaving a blunt end) or *Kpn*I (leaving a 3′ protruding end). The linearized *eGFP* empty vectors were then mixed with SYBR Green I, followed by treatment with ExoIII or T7. Each well contained 10 μg *eGFP* empty vectors and 20 units enzyme/well in a 20 μl reaction mixture. The vectors were treated with ExoIII at 37 °C or T7 at 30 °C for 50–60 min. The digestion activities of ExoIII and T7 were estimated by the reduction in fluorescence intensity, which was measured every minute with an Applied Biosystems® 7500 real-time PCR system (Thermo Fisher Scientific, MA, USA). We performed five independent experiments and averaged the resulting data. To estimate the overhang lengths of the targeting vectors that were transfected into ES cells or fertilized mouse eggs, we repeated the same experiments with those vectors.

### Zygote collection and triton treatment

To maximize the number of fertilized mouse eggs used in the neocarzinostatin (NCS) treatment, we treated the egg-donor female mice with PMSG (pregnant mare’s serum gonadotropin; Sigma-Aldrich, MO, USA) and HCG (human chorionic gonadotropin, Sigma-Aldrich, MO, USA) to induce superovulation prior to mating them with stud mice. The donor mouse strain we used was FVB/N (SLC). Isolation of fertilized eggs was performed based on a method previously described for the generation of transgenic mice^50^. The mice were sacrificed, and the cumulus masses were released into M2 media (Sigma-Aldrich) containing hyaluronidase (0.3 mg/ml, Sigma-Aldrich). M2 was used in collecting and handling the eggs for prolonged periods outside of a CO_2_ incubator. After 1 h of treatment with NCS (10 ng/ml), the fertilized eggs were fixed in 4% paraformaldehyde (PFA)/TBS and permeabilized with 0.5% Triton X-100 for 30 min. As zygotes are surrounded by the zona pellucida, a specialized extracellular matrix, we performed chemical zona pellucida thinning with acidified Tyrode’s solution (pH 2.5 ± 0.3, Sigma-Aldrich) for 30 sec to increase the efficiency of immunocytochemistry^51,52^. The zygotes were then blocked in 7% goat serum/TBS-0.1% Triton X-100, and immunostaining was performed as described below. Images were captured with a DeltaVision microscopy imaging system (GE Healthcare Life Sciences, UK).

### Immunocytochemistry

After 24 h of culture, MEF or ES cells were treated with 10 ng/ml NCS (Sigma-Aldrich) for 30 min at 37 °C just prior to fixation with 4% (w/v) PFA. Then, permeabilization was performed for 10 min with 0.2% Triton X-100, and the cells were blocked using 5% (w/v) BSA/TBS and incubated with anti-γ-H2AX (EMD Millipore, Darmstadt, Germany), Rad51 (Cell Signaling Technology, Massachusetts, USA) and 53BP1 (Novus, Missouri, USA) antibodies overnight at 4 °C. After three washes, the cells were incubated with Alexa 488-conjugated anti-rabbit IgG (Thermo Fisher Scientific) or Alexa 546-conjugated anti-mouse IgG (Thermo Fisher Scientific) for 1–2 h at room temperature. The nuclei were counterstained with 100 nM 4′,6-diamidino-2-phenylindole (DAPI) (Dojindo, Japan), images were captured using a laser scanning confocal microscope (LSM 700, Carl Zeiss, Germany).

### Gene targeting of *Rosa26* and *NudCD2* in fertilized mouse eggs by pronuclear injection

To target *Rosa26* by pronuclear injection, we modified the *Rosa26-H2B-eGFP* targeting vector that had been generated previously^53^. We first modified the original *pBluescript* vector by introducing *Asc*I and *Sfi*I sites. In this modified *pBluescript* vector, the entire insert was demarcated by *Asc*I and *Sfi*I sites at its borders, such that the entire insert could be cut out by digestion with either *Asc*I or *Sfi*I. The entire insert from *Rosa26-H2B-eGFP* was cloned into the modified *pBluescript* vector between the vector’s *Asc*I and *Sfi*I sites. Two hundred μg of targeting construct for the *Rosa26* gene was digested with *Sfi*I, followed by agarose gel electrophoresis. The insert DNA was extracted and purified from agarose gels using a Wizard SV Gel and PCR Clean-Up System (Promega, Madison, USA). Half of the extracted insert DNA was subjected to T7 treatment for 3 min at 30 °C, followed by phenol-chloroform extraction and ethanol precipitation to remove enzymes before pronuclear injection. The untreated insert was used as a control to compare the efficiency of HDR. To target *NudCD2* by pronuclear injection, we generated another targeting vector in which no neomycin cassette was inserted. We used our modified *pBluescript* vector with *Asc*I and *Sfi*I sites, and the first exon was flanked by two *loxP* sites. Two hundred μg of the targeting construct of the *NudCD2* gene was digested with *Sfi*I, followed by agarose gel electrophoresis. Insert DNA was extracted and purified from agarose gels using a Wizard SV Gel and PCR Clean-Up System (Promega). Half of the extracted insert DNA was subjected to T7 treatment for 3 min at 30 °C, followed by phenol-chloroform extraction and ethanol precipitation. The untreated insert was used as a control to compare the efficiency of HDR.

For pronuclear injection, fertilized eggs at the single-cell stage were collected from *C57BL/6* (for *Rosa26* targeting) or *FVB/NJcl* (for *NudCD2* targeting) mice. Linearized DNA constructs were injected into one of the pronuclei. The injected eggs were then transferred into the oviducts of pseudopregnant foster mice. Two weeks after birth, the mice were subjected to DNA extraction from tail clippings and the DNA was used for Southern blotting analysis using a flanking probe for either *Rosa26* or *NudCD2*. HDR events in the *Rosa26* gene were identified by a restriction polymorphism resulting from an *Eco*RI site newly introduced into the targeted allele (Supplementary Fig. 4a: WT allele 15.5 kbp, targeted allele 10.5 kbp). Similarly, HDR events in the *NudCD2* gene were determined by a newly introduced *Apa*I site in the targeted allele. (Supplementary Fig. 4e: WT allele 15.5 kbp, targeted allele 11 kbp).

### CRISPR/Cas9-mediated gene editing

For CRISPR/Cas9-mediated gene editing to introduce DNA insertion into the mouse ROSA25 gene, two crsprRNAs (crRNAs) were synthesized chemically (FASMAC, Kanagawa, Japan). One had a sequence from 5 kb upstream of the ROSA26 gene (5′-CAAACAGCUAGAUAAGUGCGGGG-3′), and the other had a sequence from the first intron of the ROSA26 gene (5′-CACAUCCAUAGUGGCUCAUUAGG-3′). The two crRNAs, trans-activating crRNA, Cas9 protein (GeneArt Platinum Cas9 Nuclease, Thermo Fisher Scientific, USA). For knock-in mouse production, the Cas9 protein, Rosa26-crRNA, tracrRNA and DNA fragment were diluted and mixed in TE buffer (10 mM Tris-HCl, 0.1 mM EDTA, pH 8.0) to concentrations of 50 ng/μl, 75 ng/μl, 75 ng/μl, and 5 ng/μl, respectively^54^. The mixture was injected into the pronuclei and cytoplasm of one-cell-stage embryos of BDF1xBDF1 mice, and the injected embryos were then transferred into the oviducts of pseudopregnant surrogate ICR female mice (Nihon SLC, Hamamatsu, Shizuoka, Japan). All animal experiments were approved by the Animal Experiment Committees of the Institute for Frontier Medical Sciences and Kyoto University.

## Supplementary information

Supplementary information
