# Supplementary material for: Enhanced homologous recombination by the modulation of targeting vector ends
Source: Sci Rep. 2020 Feb 13;10:2518. doi: 10.1038/s41598-020-58893-9 (PMC7018964; doi:10.1038/s41598-020-58893-9)
Supplement: Supplementary file 1 — Supplementary information [file 41598_2020_58893_MOESM1_ESM.docx]

**Supplementary Information**

**Enhanced homologous recombination by the modulation of targeting vector ends**

Shinji Hirotsune^1^*, Hiroshi Kiyonari^2^, Mingyue Jin^1^, Kanako Kumamoto^1^, Kayo Yoshida^3^, Miki Shinohara^4,5^, Hitomi Watanabe^6^, Anthony Wynshaw-Boris^7^ and Fumio Matsuzaki^8^

^1^Department of Genetic Disease Research, Osaka City University, Graduate School of Medicine, Asahi-machi 1-4-3, Abeno, Osaka 545-8585, Japan

^2^Animal Resource Development Unit, Genetic Engineering Team, Division of Bio-Function Dynamics Imaging, RIKEN Center for Life Science Technologies, 2-2-3 Minatojima-Minamimachi, Chuou-ku, Kobe 650-0047, Japan

^3^Laboratory Animal Science, Osaka City University, Graduate School of Medicine, Asahi-machi 1-4-3, Abeno, Osaka 545-8585, Japan

^4^Institute for Protein Research, Osaka University 3-2 Yamadaoka, Suita, Osaka　565-0871 Japan

^5^Present address; Faculty of Agriculture, Department of Advanced Bioscience, Kindai University, 3327-204 Nakamachi, Nara-city, Nara 631-8505, Japan

^6^Laboratory of Integrative Biological Science, Institute for Frontier Life and Medical Sciences, Kyoto University, Kyoto 606-8507, Japan

^7^Department of Genetics and Genome Sciences, Case Western Reserve University, School of Medicine, University Hospitals Case Medical Center 10900 Euclid Avenue, BRB731 Cleveland, Ohio 44106-4955, USA

^8^RIKEN Center for Developmental Biology, 2-2-3 Minatojima-Minamimachi, Chuou-ku, Kobe 650-0047, Japan

*Corresponding author

E-mail address: [shinjih@med.osaka-cu.ac.jp](mailto:shinjih@med.osaka-cu.ac.jp)


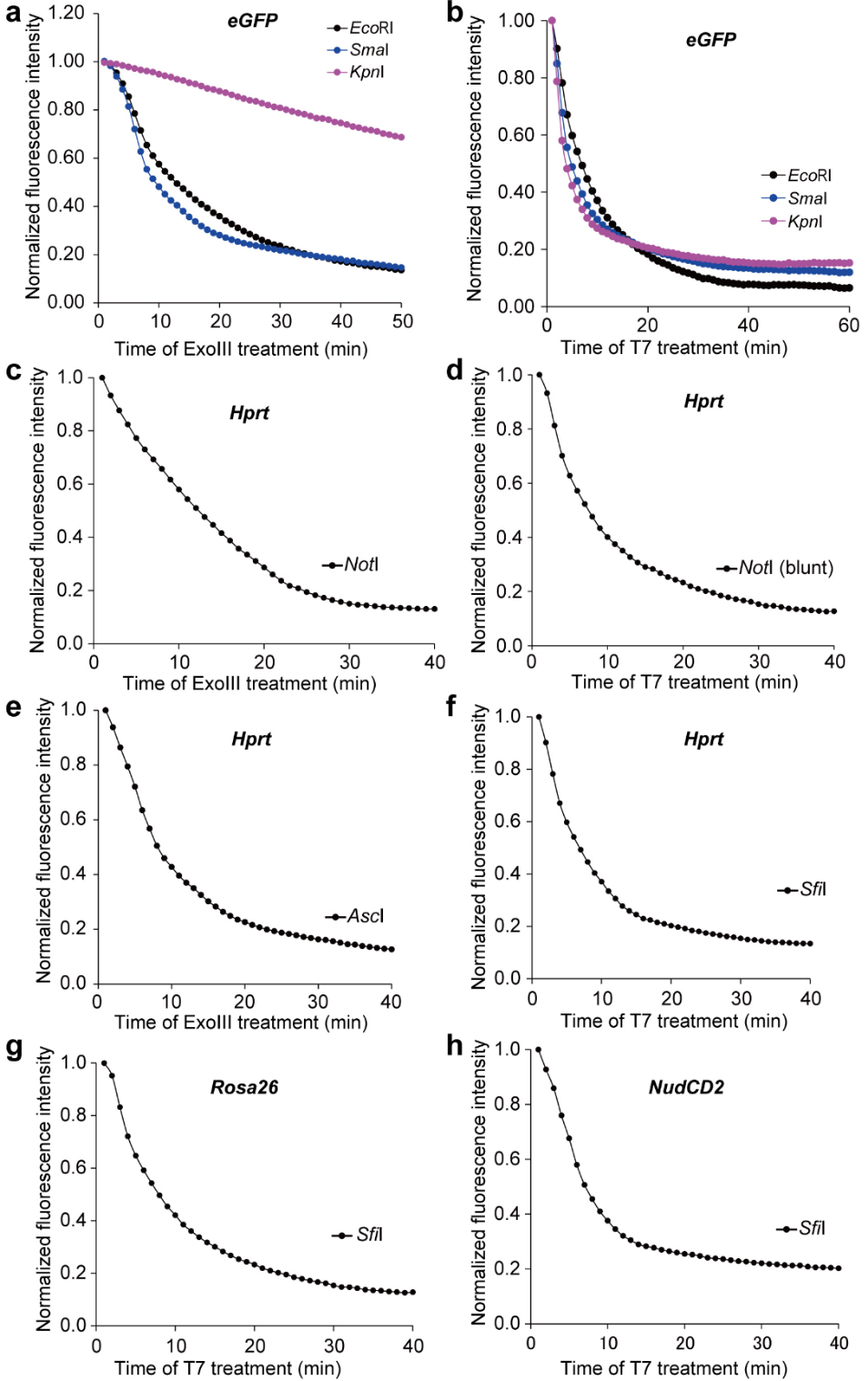


**Supplementary Figure 1. Examination of ExoIII and T7 activity using SYBR Green.** (**a**,**b**) The eGFP empty vector was linearized by *Eco*RI, *Sma*I or *Kpn*I to produce 5’ protruding ends, blunt ends or 3’ protruding ends, respectively. After intercalation of SYBR Green, each linearized plasmid was treated with ExoIII (a) or T7 (b). The X-axis indicates elapsed time (min), and the Y-axis indicates the relative intensity of SYBR Green. The starting points are normalized as 1.0. Note: the eGFP empty vector carrying 3’ protruding ends by *Kpn*I was relatively resistant to *Exo*III digestion, whereas T7 was highly active at all types of restriction sites. The eGFP empty vector is 4.7 kbp in length. According to the reduction in fluorescence intensity, ExoIII and T7 remove nucleotides at 100 bp/min and 150 bp/min, respectively. (**c-h**) Enzymatic activities of ExoIII and T7 measured on the targeting vectors used in this study. The *Hprt* targeting vectors were treated by ExoIII after *Not*I digestion (c, also see Fig. 1b), by T7 after *Not*I digestion followed by a fill-in reaction using *Klenow* enzyme (d, also see Fig. 1c), by ExoIII after *Asc*I digestion (e, also see Fig. 1e), or by T7 after *Sfi*I digestion (f, also see Fig. 1f). (**g**,**h**) The targeting vector used for the *Rosa26* locus (g) or *NudcCD2* (h); these targeting vectors were treated by *Sfi*I and T7 (also see Fig. 4a,b). The enzymatic activities measured in (c-h) were reproducible, as estimated in (a,b).


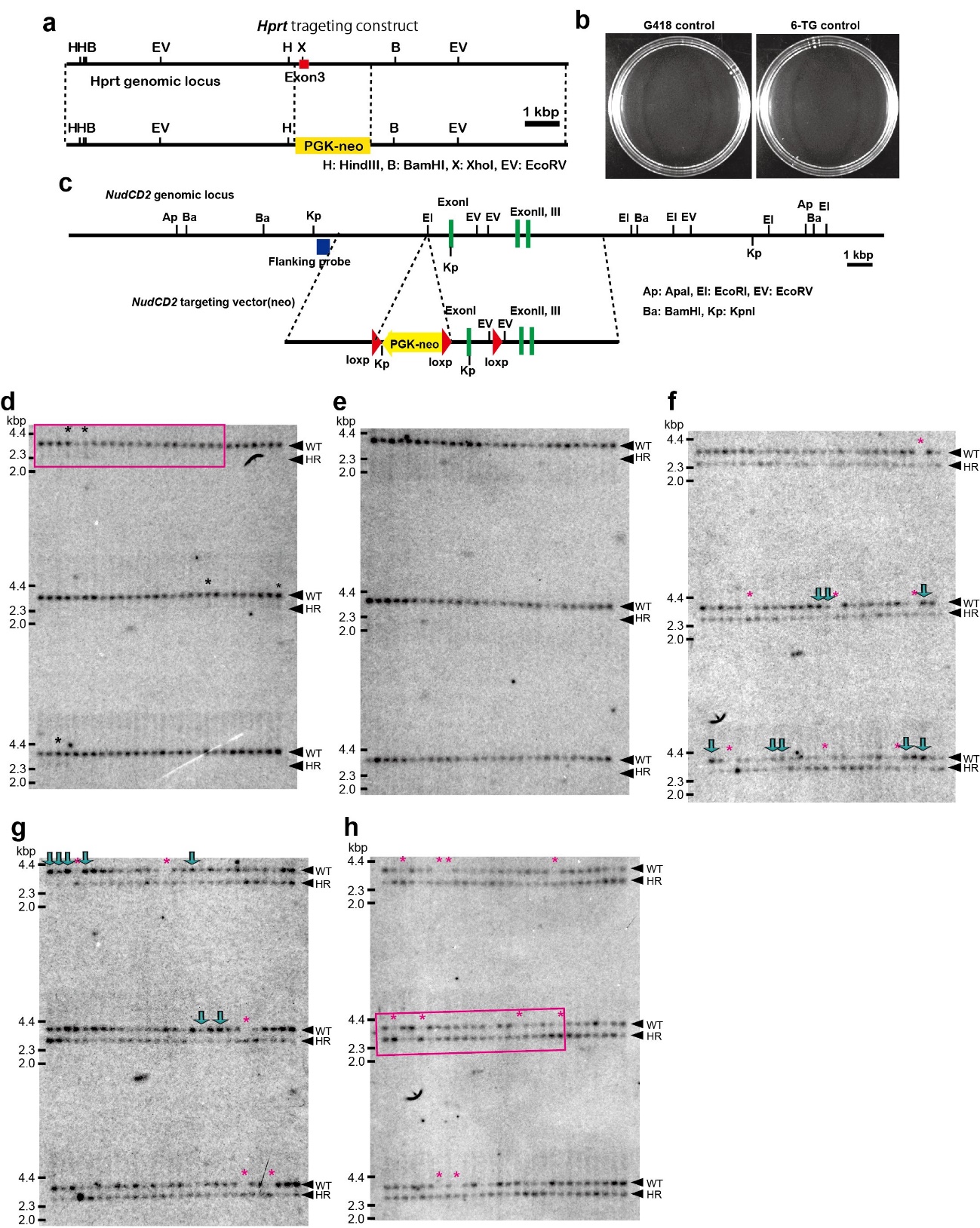


**Supplementary Figure 2. Experimental design for homologous targeting of the mouse *Hprt* gene.** (**a**) A map of the replacement vector for *Hprt*. The targeting vector contains the neomycin cassette *PGK-neo*, which replaced exon 3 and the neighboring introns of the *Hprt* gene. The vector was made from isogenic *129/Sv* DNA. (**b**) Without transfection of the targeting vector, only a few colonies were present after the selection of G418 or 6-TG. Inactivation of *Hprt* renders the cells resistant to 6-TG, which kills *Hprt*^+^ cells. (**c**) A map of the replacement vector for *NudCD2*. The targeting vector contains the neomycin cassette *PGK-neo*, which was inserted in front of the first exon of the *NudCD2* gene. The neomycin cassette was flanked by two *loxP* sequences. The third *loxP* site was inserted into intron 1. HR-based replacement produces a novel *Kpn*I site, which was used as the flanking probe for detection of the HR event by Southern blotting using G418-resistant clones. (**d**,**e**) Simply linearized targeting vector for *NudCD2* detected by Southern blotting. Black asterisks indicate homologous recombinants. (**f-h**) Efficient HR obtained by T7-treated *NudCD2*. Arrowheads in (d-h) indicate the WT allele (3.1 kbp) and targeted allele (2.5 kbp), as indicated. Note: after treatment with T7, the majority of clones contained the targeted allele. In particular, clones homozygous for the target allele were also present (magenta asterisks). Arrows indicate clones lacking the targeted allele. Rectangle surrounded area in (d) and (h) were shown in Fig. 2b.


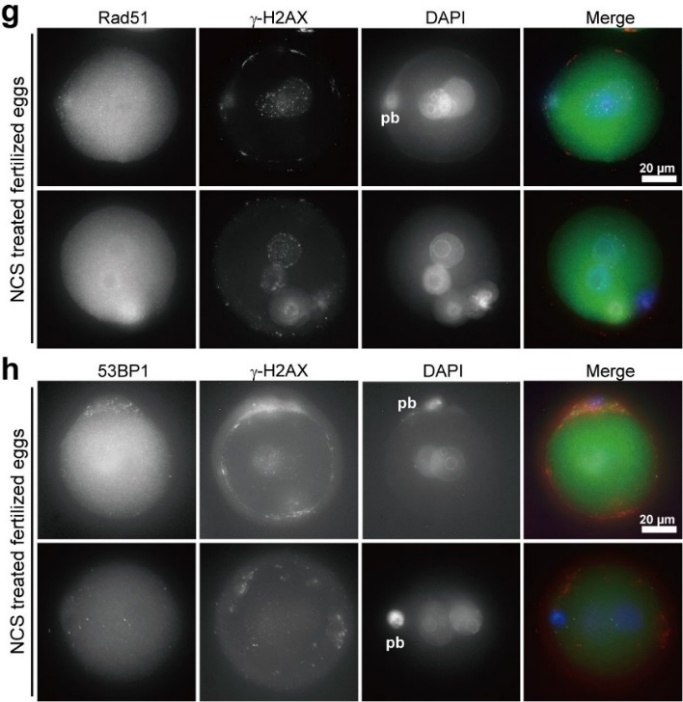

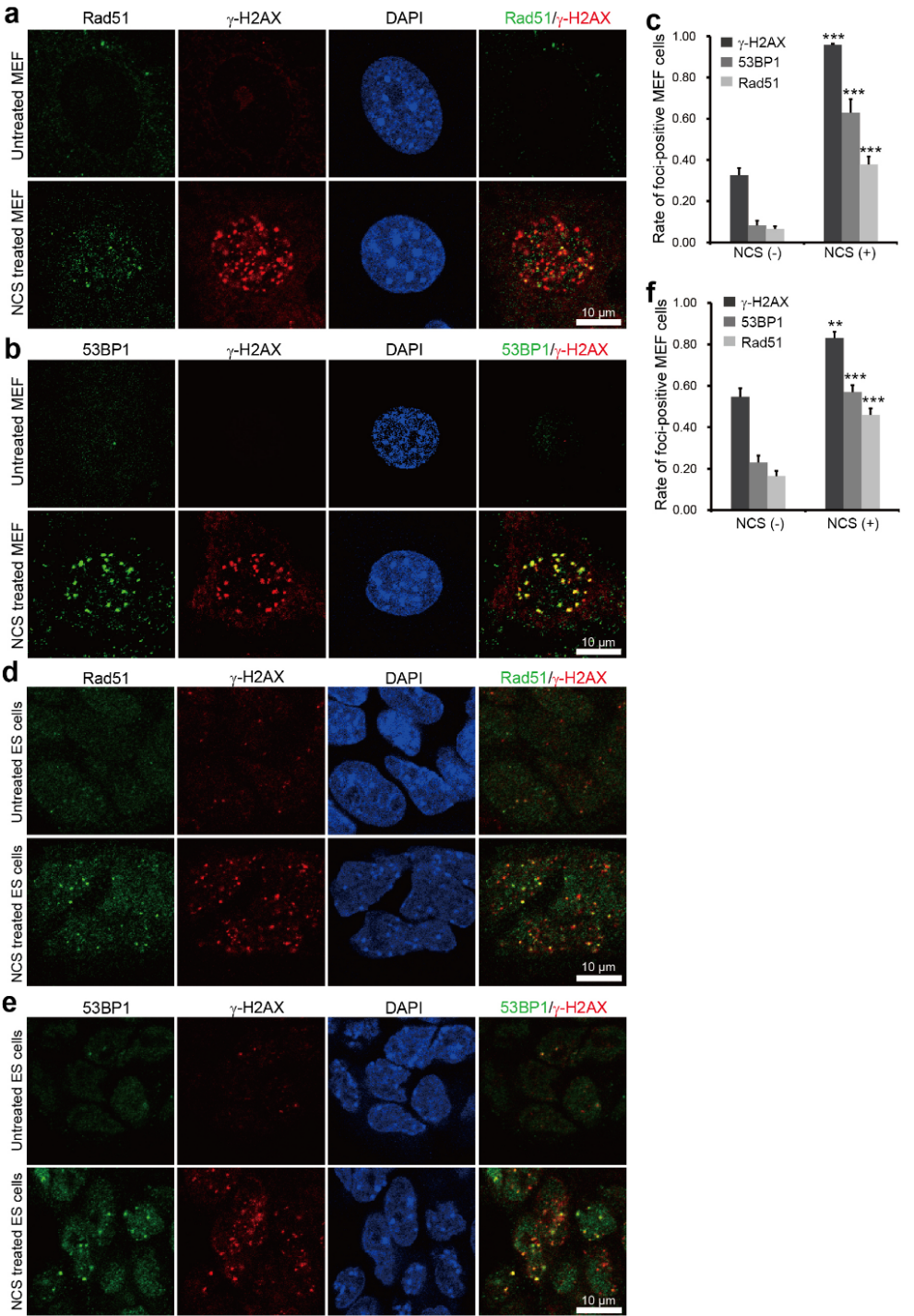


**Supplementary Figure 3. Examination of DNA repair after introduction of DSB in MEF cells, ES cells and mouse eggs.** DNA repair was probed with increased γ-H2AX, Rad51 and 53BP1 expression in MEF and ES cells, as well as in fertilized mouse eggs after NCS treatment. (**a**,**b**,**d**,**e**,**g**,**h**) MEF cells (a,b), ES cells (d,e) or fertilized mouse eggs (g,h) were treated with 10 ng/ml NCS, and their DNA damage was probed with antibodies against γ-H2AX, Rad51 and 53BP1, as indicated. (**c**,**f**) MEF cells (c) or ES cells (f) containing more than 5 foci were used for statistical analyses, and the graphs are shown in (c) (*n* = 484 for untreated MEF cells as control, *n* = 538 for NCS treated MEF cells) and (f) (*n* = 486 for untreated ES cells as control, *n* = 532 for NCS treated ES cells). *P*-values were calculated using Student’s *t*-test, mean ± s.e., ***P* < 0.01, ****P* < 0.001. Scale bar: 10 μm in (a,b,d,e); 20 μm in (g,h). pb: polar body.

**
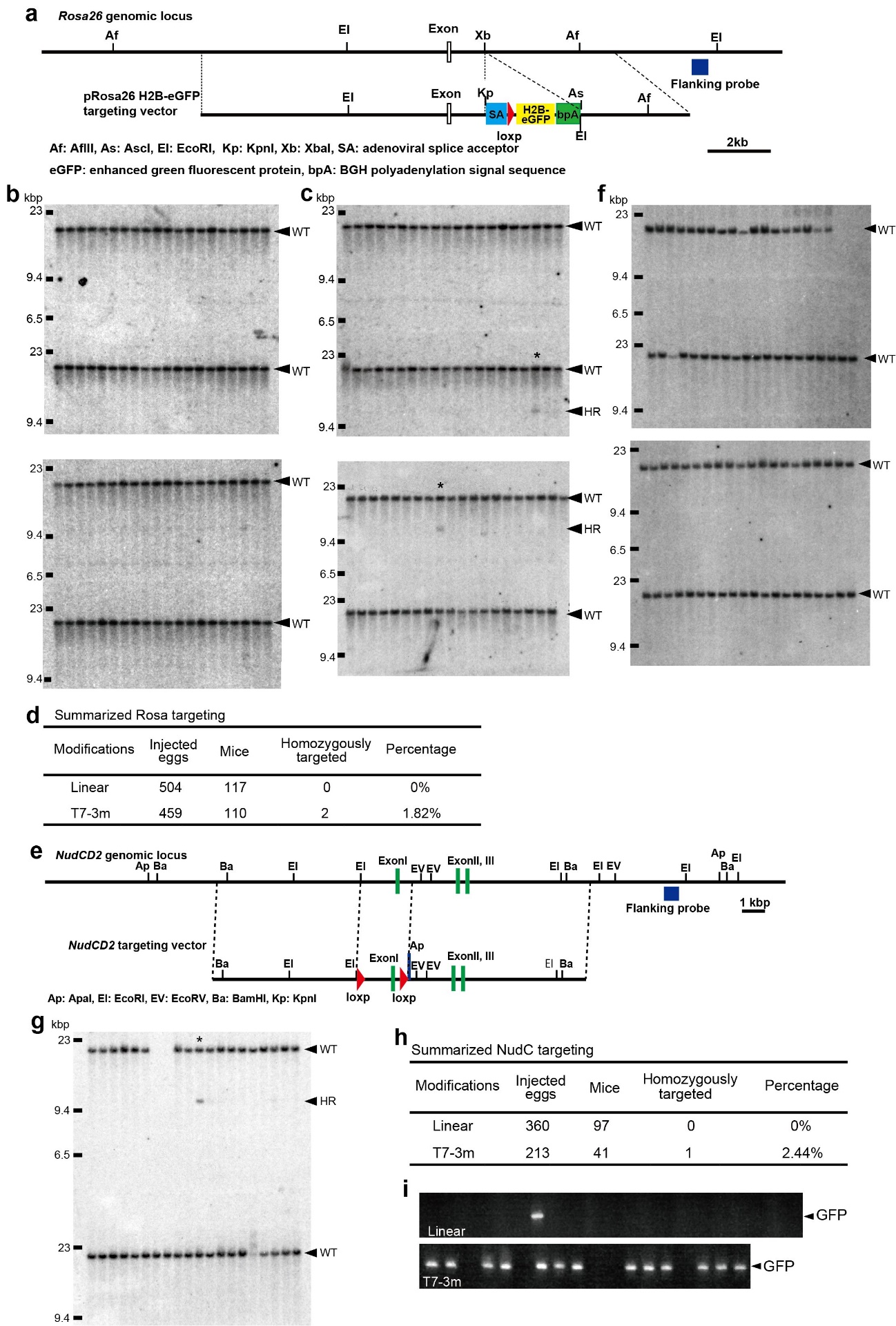
**

**Supplementary Figure 4. Gene targeting of *Rosa26* and *NudCD2* investigated by Southern blotting analyses.** (**a**) A map of the replacement vector used to target *Rosa26* by pronuclear injection. HR-mediated replacement introduces a new *Eco*RI site for the Southern blotting analysis. (**b**) Southern blotting analysis of the pups produced by targeting the *Rosa26* locus with pronuclear injection using the unmodified vector. Polymorphisms after *Eco*RI digestion were detected by the flanking probe, as indicated in (a). (**c**) Southern blotting analysis of the pups produced by targeting the *Rosa26* locus with pronuclear injection using the modified vector. Polymorphisms after *Eco*RI digestion were detected by the flanking probe, as indicated in (a). (**d**) Summary of Southern blotting analysis of the pups produced by targeting the *Rosa26* locus with pronuclear injection. (**e**) A map of the replacement vector used to target *NudCD2* by pronuclear injection. HR-mediated replacement introduces a new *Apa*I site for Southern blotting analysis. (**f**) Southern blotting analysis of the pups produced by targeting *NudCD2* with pronuclear injection using the unmodified vector. Polymorphisms after *Apa*I digestion were detected by the flanking probe, as indicated in (e). (**g**) Southern blotting analysis of the pups produced by targeting *NudCD2* with pronuclear injection after modification. Polymorphisms after *Apa*I digestion were detected by the flanking probe, as indicated in (e). The asterisks in (c) and (g) indicate homologous recombinants. A *Hind*III digest of lambda DNA was used as a size marker in the Southern blotting analyses (left side). (**h**) Summary of Southern blotting analysis of the pups produced by targeting the *NudCD2* locus with pronuclear injection. (**i**) PCR confirmation of the presence of a targeting vector using the vector-specific primers (GTTGTTATCAGTAAGGGGAGCTGCAGT and GCGATGCGCTCGAA AATGTCGTTC).
